# Role of CDH23 as a prognostic biomarker and its relationship with immune infiltration in acute myeloid leukemia

**DOI:** 10.1186/s12885-022-09532-1

**Published:** 2022-05-21

**Authors:** Jiao Yang, Fei Lu, Guangxin Ma, Yihua Pang, Yanan Zhao, Tao Sun, Daoxin Ma, Jingjing Ye, Chunyan Ji

**Affiliations:** 1 Department of Hematology, Qilu Hospital, Cheeloo College of Medicine, Shandong University, Jinan, 250012 Shandong China; 2 Hematology and Oncology Unit, Department of Geriatrics, Qilu Hospital of Shandong University, Jinan, China

**Keywords:** CDH23, AML, Prognosis, Immune infiltration

## Abstract

**Background:**

Cadherin-23 (CDH23) plays an important role in intercellular adhesion and is involved in the progression of several types of cancer. However, the biological functions and effect of CDH23 expression on the prognosis of patients with acute myeloid leukemia (AML) are unexplored. Herein, we aim to characterize the role and molecular functions of CDH23 in AML.

**Methods:**

We downloaded the transcriptomic profiles and clinical data from the Cancer Genome Atlas and Beat AML trial. The expression level of *CDH23* was assessed using Gene Expression Profiling Interactive Analysis (GEPIA). Kaplan-Meier survival analysis was used to assess prognostic value of *CDH23*. Correlation and biological function analyses were performed using LinkedOmics and GeneMANIA. Relationship of CDH23 with immune infiltration level was determined using Tumor Immune Estimation Resource (TIMER).

**Results:**

We found that the *CDH23* expression was aberrantly upregulated in patients with AML and could be used as an independent risk factor of overall survival using Cox multivariate analysis. Notably, we observed a negative correlation between CDH23 expression and immune cell infiltration abundance by calculating the immune and stromal scores. In addition, functional enrichment analysis established that CDH23 plays a crucial role in tumor immunity.

**Conclusions:**

Our findings indicate that upregulated *CDH23* expression corresponds to decreased overall survival of patients with AML. CDH23 may be involved in mediating tumor immune environment, and this highlights the potential of CDH23 as a therapeutic target in AML.

**Supplementary Information:**

The online version contains supplementary material available at 10.1186/s12885-022-09532-1.

## Background

Acute myeloid leukemia (AML) is an aggressive clonal hematopoietic malignancy characterized by an accumulation of immature progenitor cells of the myeloid lineage in the bone marrow that leads to inhibition of normal hematopoietic cell proliferation [1–3]. Most AML patients can respond to standard chemotherapy. However, many patients who present drug resistance or have short remission durations have median overall survival (OS) of 3-7 months [4, 5]. In addition, the prognosis of older patients with AML is still dismal despite recent improvements in therapeutic and diagnostic strategies [6].

The cadherin superfamily plays important roles in a plethora of biological processes and diseases, including signal transduction, self-recognition, and tumor suppression, by regulating intercellular adhesion and cell-cell recognition in a Ca2^+^-dependent manner [7–11]. Cadherin-23 (CDH23) is defined as an atypical cadherin and dysregulation of CDH23 expression remarkably disrupts homotypic and heterotypic adhesions and increases the metastatic potential of various tumors [12–17]. Furthermore, the downregulation of CDH23 expression was significantly association with poorer outcome in patients with diffuse large B cell lymphoma [18]. However, the role of CDH23 in AML remains unknown.

In this study, we investigated the expression profile and biological functions of CDH23 in AML and further analyzed the association between CDH23 expression and the AML tumor immune microenvironment using systematic bioinformatics analysis.

## Methods

### Data collection and processing

Data of 145 bone marrow tissue samples from patients with AML were obtained from the Cancer Genome Atlas (TCGA) (https://portal.gdc.cancer.gov/repository), currently the largest cancer multi-omics information database [19]. Patients lacking survival time and/or status were excluded from the analysis. In TCGA, cytogenetic risks were categorized as poor, intermediate, and favorable risk according to National Comprehensive Cancer Network guideline.

Dato of 139 *de novo* AML patients were included from Beat AML trial and used as a validation cohort with complete clinical information. The aim of the Beat AML trial is to publicly provide cytogenetic and mutational data before and after treatment [20].

### GEPIA

Gene Expression Profiling Interactive Analysis (GEPIA) (http://gepia.cancer-pku.cn/index.html) is an analysis tool that contains RNA sequencing expression data of 9,736 tumors and 8,587 normal samples from TCGA and the GTEx projects [21]. In this study, we utilized the “Single Gene Analysis” module to perform a differential mRNA expression and prognostic analyses of CDH23 expression in AML patients and healthy donors. The *P* value cutoff was set at 0.05. Prognostic analysis was performed using a Kaplan-Meier curve.

### LinkedOmics

LinkedOmics (http://www.linkedomics.org/) is a publicly accessible portal that includes multi-omics data from all 32 TCGA cancer types. The web application has three analytical modules: LinkFinder, LinkInterpreter, and LinkCompare. We used the “LinkFinder” module to investigate the transcriptional factor target enrichment of CDH23. The analysis results can be visualized using scatter, box, or Kaplan-Meier plots. To obtain biological insights from the association results, the LinkInterpreter module performs gene set enrichment analysis based on Gene Ontology (GO), Kyoto Encyclopedia of Genes and Genomes (KEGG), and other functional categories [22]. The enriched pathways were finally visualized using the dotplot and emapplot functions.

### TIMER

Tumor Immune Estimation Resource (TIMER) (https://cistrome.shinyapps.io/timer/) is a comprehensive resource that provides systematic analysis of immune infiltration [23]. In our study, we used the “Estimation” module to evaluate the correlation between CDH23 expression level and immune infiltration in the AML dataset from TCGA using several computational algorithms. Immune cell scores were described by the immune and stromal scores. Different immune cell types were investigated, including macrophages, neutrophils, and B, natural killer, CD8^+^T, CD4^+^T, and dendritic cells. The association of gene expression level with immune cell scores was considered significant when *P*<0.05.

### GeneMANIA

A protein-protein interaction (PPI) network was constructed using GeneMANIA (http://www.genemania.org/), a useful web resource that can explore the potential functions of selected genes and construct a PPI network. Association data including protein and genetic interactions, pathways, co-expression, co-localization, and protein domain similarity can be investigated by using GeneMANIA [24].

### Statistical analyses

Statistical analyses were conducted using R language (version 4.0.1). Differences between two groups were evaluated using the Mann-Whitney U-test. OS analysis was performed using the Kaplan-Meier method and compared using log-rank tests. Univariate and multivariate cox analysis was conducted using logistic model. *P*<0.05 indicated statistically significant differences.

## Results

### Association between clinical characteristics and CDH23 expression in AML patients

Data of 145 and 139 adult patients with AML from TCGA and Beat AML, respectively, were analyzed in present study. The clinicopathologic features of AML patients between high and low *CDH23* expression groups divided by the median value are shown in Table 1. We observed that there were no significant differences between two groups in gender, leukocyte count, hemoglobin value, platelet count, blast percentage, and genetic mutations (*FLT3, NPM1, DNMT3A, IDH1, IDH2, RUNX1, NRAS, CEBPA, TET2, ASXL1, TP53)* or fusions (Additional file 1: Fig. S1). However, the age and cytogenetic risk group were correlated with the *CDH23* expression value in TCGA cohort.

**Table 1:** Clinicopathologic characteristics of patients in two cohorts

		Low CDH23 (%)	High CDH23 (%)	*P*-value
**Training cohort**				
** (TCGA-LAML, *N* = 145)**		**72**	**73**	
Age	<60y	50 (69.44)	35 (47.95)	0.013
	≥60y	22 (30.56)	38 (52.05)	
Gender	female	36 (50)	31 (42.47)	0.457
	male	36 (50)	42 (57.53)	
Clinical characteristics (mean ± sd)	Blast count (%)	45.39 ± 34.93	31.14 ± 27.6	0.006
	Leukocyte (x10^9)	33.99 ± 47.66	36.75 ± 39.14	0.703
	Hemoglobin (g/L)	9.62 ± 1.53	9.53 ± 1.41	0.692
	Platelet count (x10^9)	57.21 ± 50.8	69.73 ± 48.84	0.130
Cytogenetic risk	Poor	14 (20)	12 (16.44)	< 1e-04
	Intermediate	31 (44.29)	57 (78.08)	
	Favorable	25 (35.71)	4 (5.48)	
Mutations	*FLT3*	20 (28.17)	21 (29.17)	1.000
	*NPM1*	16 (22.54)	26 (36.11)	0.109
	*DNMT3A*	16 (22.54)	20 (27.78)	0.596
	*IDH1*	11 (15.49)	4 (5.56)	0.095
	*IDH2*	7 (9.86)	8 (11.11)	1.000
	*NRAS*	4 (5.63)	6 (8.33)	0.760
	*CEBPA*	9 (12.68)	3 (4.17)	0.125
	*RUNX1*	5 (7.04)	9 (12.5)	0.414
	*TET2*	9 (12.68)	6 (8.33)	0.565
	*ASXL1*	1 (1.41)	2 (2.78)	1.000
	*TP53*	3 (4.23)	7 (9.72)	0.336
**Validation cohort**				
** (Beat-AML, *N* = 139)**		**69**	**70**	
Age	<60y	36 (52.17)	35 (50)	0.931
	≥60y	33 (47.83)	35 (50)	
Gender	female	36 (52.17)	27 (38.57)	0.149
	male	33 (47.83)	43 (61.43)	
Cytogenetic risk	Poor	15 (32.61)	15 (35.71)	0.413
	Intermediate	22 (47.83)	23 (54.76)	
	Favorable	9 (19.57)	4 (9.52)	
Mutation	*FLT3*	19 (27.54)	14 (20.29)	0.424
	*NPM1*	19 (27.94)	12 (17.39)	0.203
	*CEBPA*	1 (1.45)	0	0.994
Fusions	CBFB-MYH11; inv (16)	2 (2.9)	2 (2.86)	1.000
	MLLT3-KMT2A; t (9;11)	0	5 (7.14)	0.071
	RUNX1-RUNX1T1; t (8;21)	0	2 (2.86)	0.482

### Aberrant expression and prognostic value of CDH23 in AML patients

**Fig. 1:**
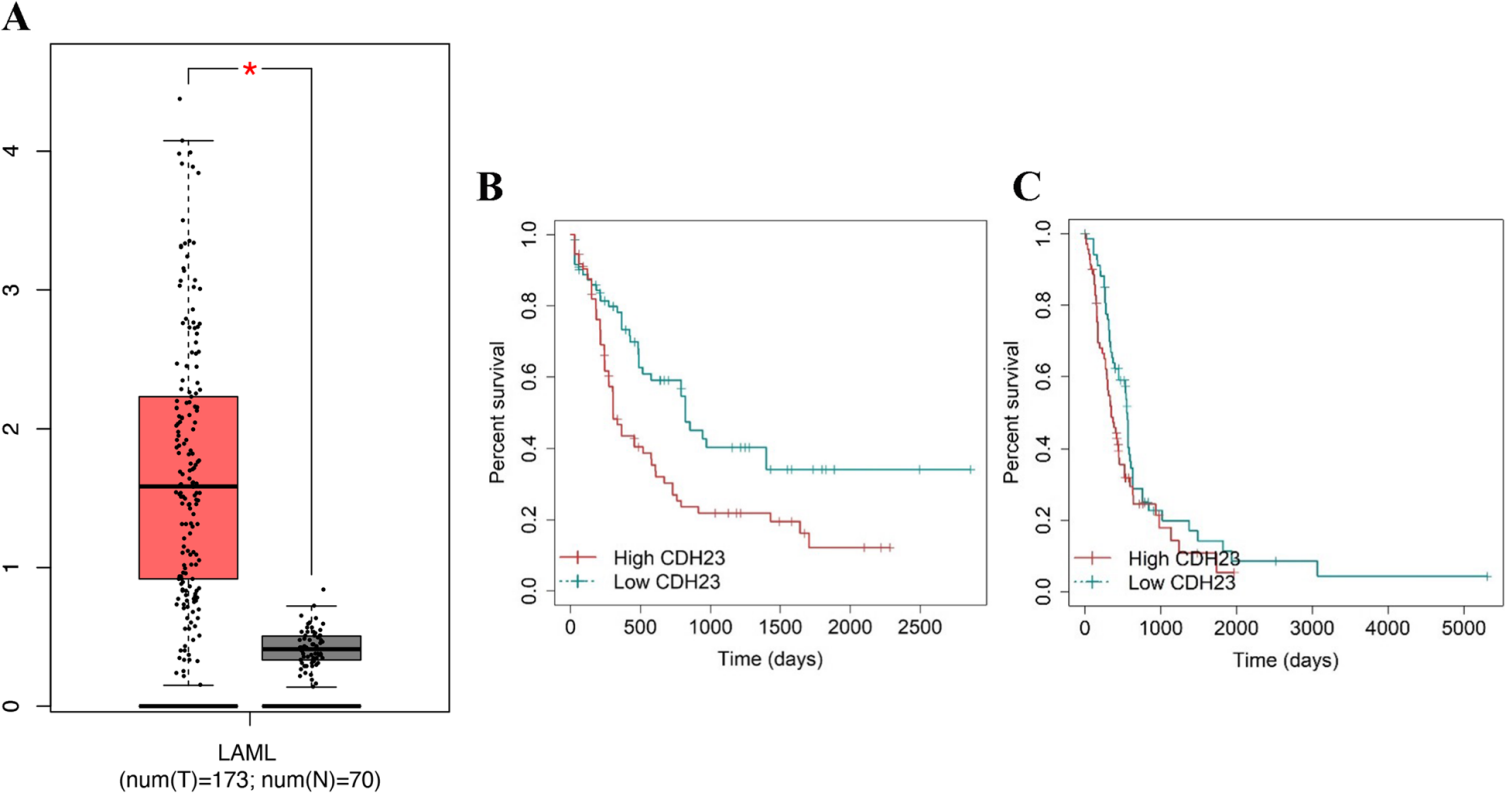
The transcription level and prognostic feature of CDH23 in AML. **A** The expression status of CDH23 gene in AML patients and paired normal bone marrow samples was analyzed by GEPIA. **B-C** Kaplan-Meier curves of CDH23 in the TCGA-LAML cohort and Beat AML cohort, respectively

As shown in Fig. 1A, we utilized the GEPIA dataset to analyze *CDH23* expression level in AML patients. *CDH23* mRNA expression was significantly higher in AML tissues compared to that in the corresponding healthy bone marrow samples, as seen from TCGA and GTEx data. We then investigated whether *CDH23* level was predictive of survival of patients with AML. In total, 145 patients from TCGA dataset were divided into high and low *CDH23* expression groups by the median mRNA level. Kaplan-Meier survival analysis demonstrated that high *CDH23* mRNA levels were associated with shorter OS of patients with AML (Hazard ratio=1.9, *P*=0.01, Fig. 1B). A total of 139 patients from the Beat AML dataset were stratified into the high and low risk groups according to the median value of *CDH23* expression. And results were consistent with those of the training set, suggesting that upregulated *CDH23* expression was as a risk prognostic factor and predicted poorer outcome in the validation cohort (HR=1.9, *P*=0.055, Fig. 1C).

### Univariate and multivariate Cox analyses of the two cohorts

The univariate and multivariate Cox analyses for *CDH23* expression and other prognostic risk factors were studied in the two cohorts (Table 2). Univariate analysis showed that besides high *CDH23* expression level, age and cytogenetic risk group greatly correlated with the OS of patients. Furthermore, when we subsequently adjusted these clinical factors in multivariate analysis, high *CDH23* expression was still identified as an independent prognostic factor for the OS of AML patients.

**Table 2:** Univariable and multivariable Cox regression analysis of OS in two cohorts

data set	variables	univariable Cox regression			multi-variable Cox regression		
		*β*	SE	*P*	*β*	SE	*P*
TCGA-LAML	age_discrete	0.985	0.214	<0.001	0.863	0.222	<0.001
	cytogenetics_risk_category	-0.576	0.172	0.001	-0.374	0.184	0.043
	CDH23	0.180	0.071	0.011	0.156	0.077	0.042
	gender	-0.107	0.211	0.613	-	-	-
	bone_marrow_blast_percent	0.003	0.003	0.355	-	-	-
	leukocyte	0.004	0.002	0.101	-	-	-
	hemoglobin	0.063	0.07	0.369	-	-	-
	platelet	0.001	0.002	0.724	-	-	-
Beat-AML	age_discrete	0.530	1.700	0.007	0.545	1.724	0.006
	CDH23	0.137	1.146	0.056	0.142	1.152	0.046

### Correlation gene expression analysis in AML patients

**Fig. 2:**
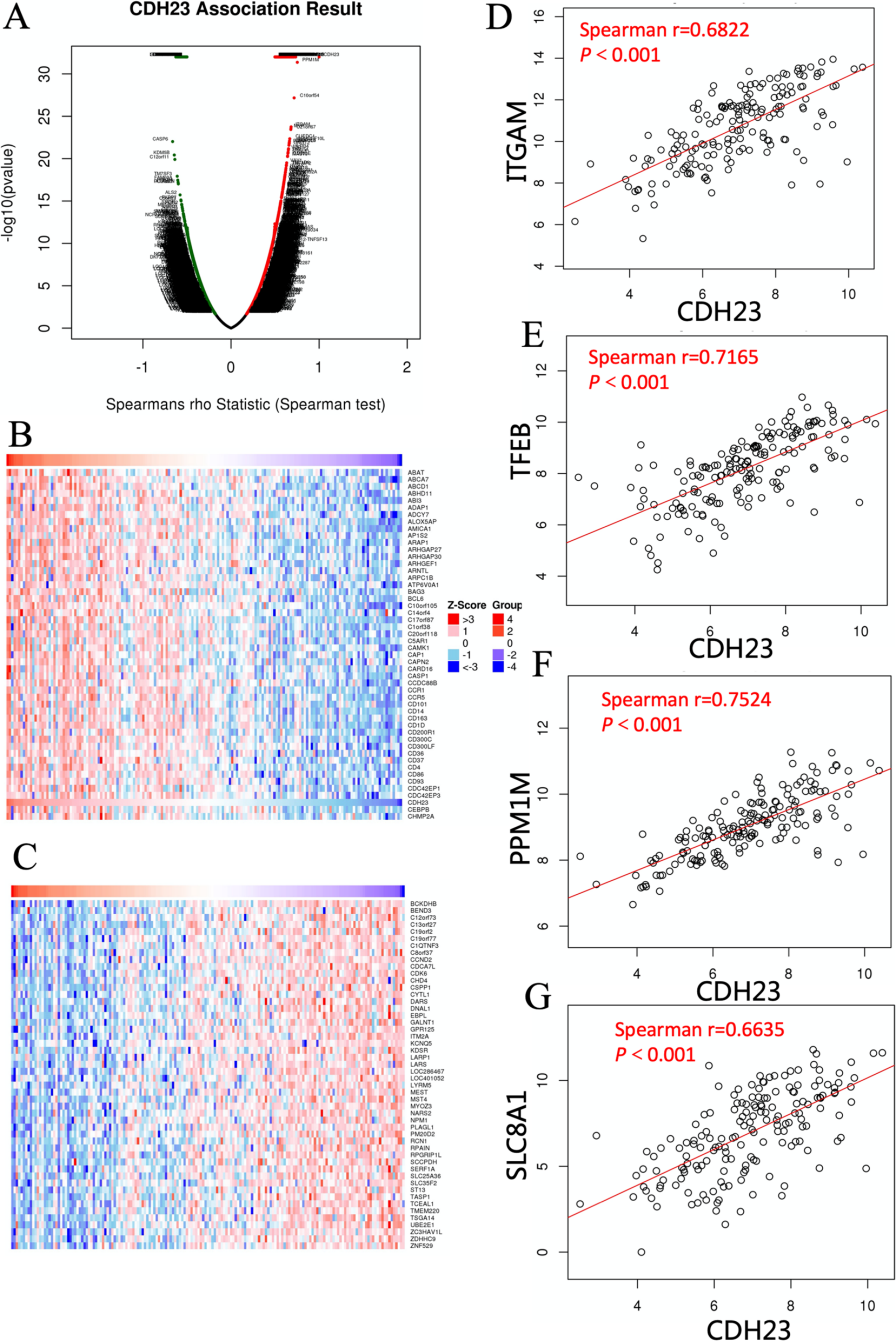
Correlated genes analysis of CDH23 in TCGA-LAML cohort (LinkedOmics). **A** The volcano plots show the correlated genes of CDH23. **B-C** The heatmaps demonstrate positively and negatively differential expression genes, respectively. **D-G** The scatter plots show Spearman-correlation of CDH23 expression with ITGAM, TFEB, PPM1M, SLC8A1

**Fig. 3:**
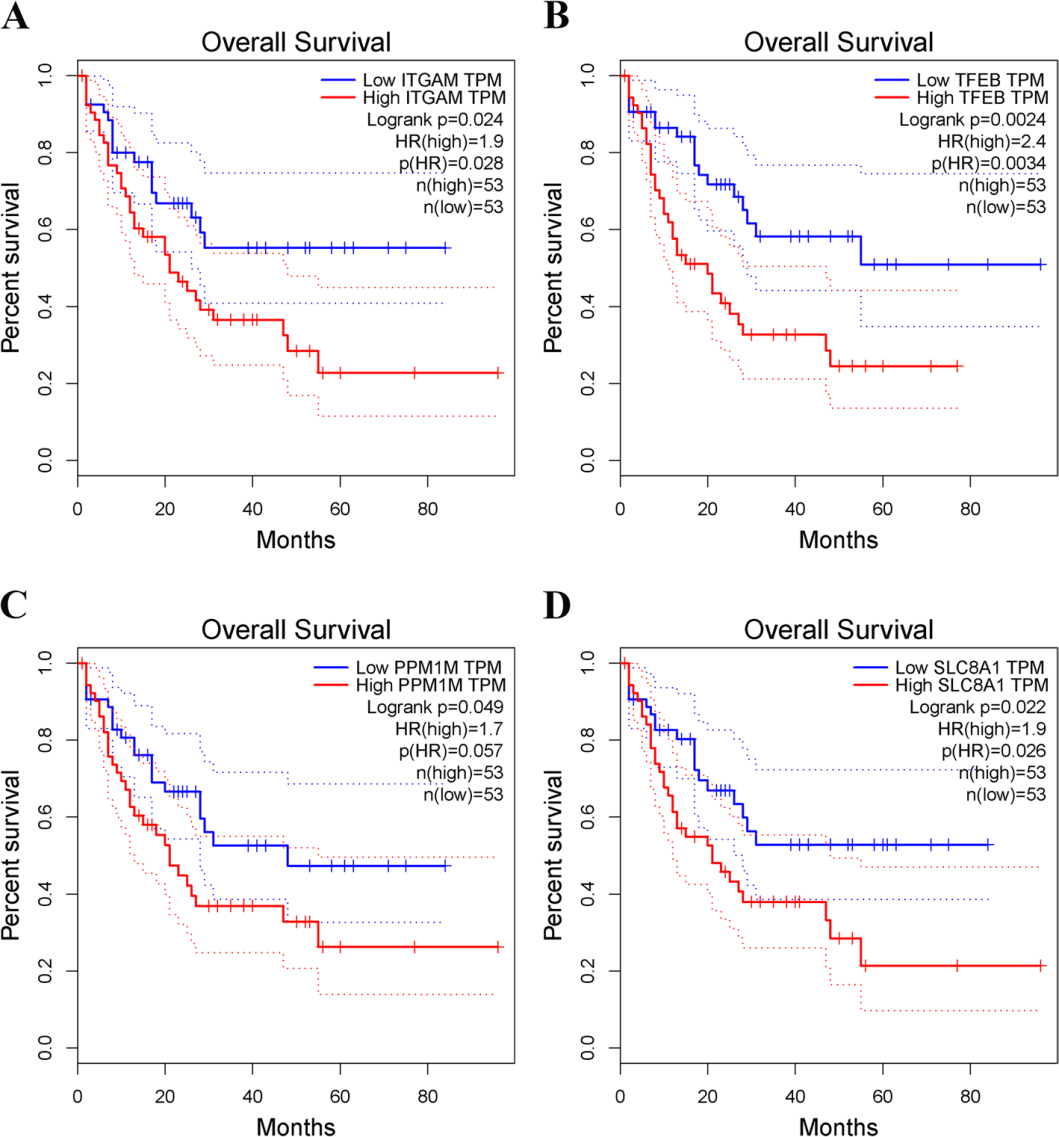
The prognostic value of genes significantly associated with CDH23 in TCGA-LAML (GEPIA). **A** The OS curves of high and low ITGAM expression in AML patients. **B** The OS curves of high and low TFEB expression in AML patients. **C** The OS curves of high and low PPM1M expression in AML patients. **D** The OS curves of high and low SLC8A1 expression in AML patients

To illustrate the potential mechanisms and functions of *CDH23* in AML, we used the LinkedOmics database for correlation analysis between *CDH23* and various genes. The results in Fig. 2A-C are presented as heat and volcano maps of the top 50 genes that are either positively or negatively correlated with *CDH23* expression. *CDH23* was positively correlated with *PPM1M*, *C10orf105*, *TFEB*, *FGD2*, and *IGF2R*. By contrast, *CDH23* demonstrated a significant negative correlation with *CASP6*, *KDM5B*, *KCNQ5*, and *SCCPDH*. We chose the most frequently altered neighboring genes, including *ITGAM* (Spearman’s correlation: 0.6822, *P*=1.765e-24), *TFEB* (Spearman’s correlation: 0.7165, *P*=0), *PPM1M* (Spearman’s correlation: 0.7524, *P*=4.274e-32), and *SLC8A1* (Spearman’s correlation: 0.6635, *P*=0), to conduct a correlation analysis using LinkedOmics (Fig. 2D-G). As shown in Fig. 3, we further analyzed the prognostic value of these four genes in AML. The results demonstrated that overexpression of *ITGAM* was highly associated with poor prognosis. Similarly, high *TFEB*, *PPM1M*, and *SLC8A1* mRNA expression corresponded to poor OS.

### Effect of CDH23 expression on immune cell infiltration and tumor microenvironment (TME) in AML patients

**Fig. 4:**
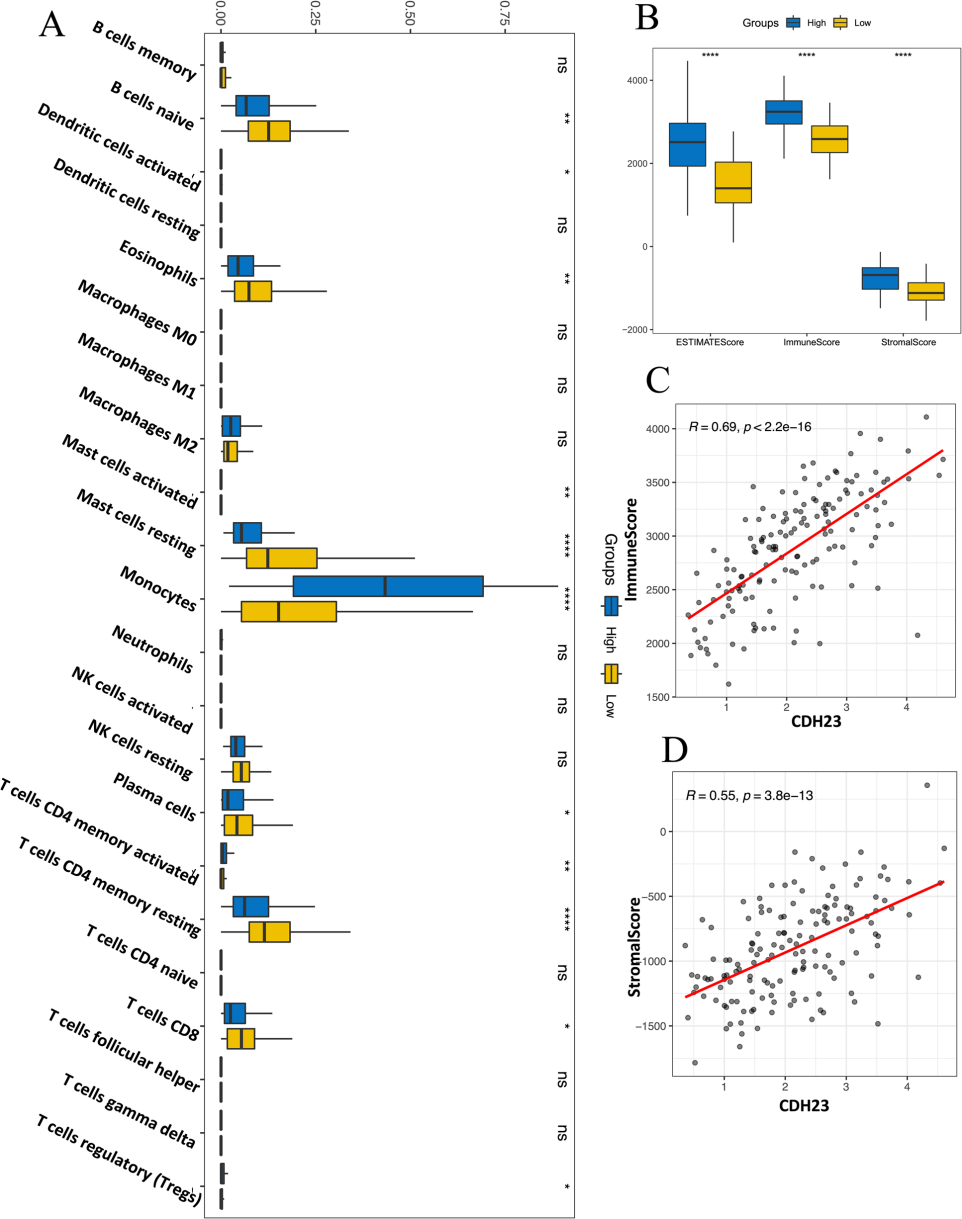
Correlation analysis between CDH23 expression in AML and tumor immune infiltration and tumor microenvironment (TIMER). **A** Different algorithms were conducted to investigate the potential correlation between the expression level of CDH23 and the various immune cell infiltration level in AML. *, *P*<0.05; **, *P*<0.01; ***, *P*<0.001; ****, *P*<0.0001. **B-D** Correlation analysis between CDH23 expression in AML and immune score and stromal score

Tumor-infiltrating immune cells (TIICs) are a major component of the TME and are involved in the occurrence, progression, and metastasis of cancer. Tumor-infiltrating lymphocyte grade is a powerful independent predictor of sentinel lymph node status and clinical survival in some cancer types [25, 26]. To explore the relationship between CDH23 expression and immune cell infiltration, we embarked on a comprehensive analysis using the TIMER database with the sequencing data of AML patients from TCGA. The online database stratified patients with AML into high and low CDH23 expression groups by the median value. Subsequently, we used several computational algorithms to estimate the abundance of various TIICs (B cells, CD4^+^T cells, CD8^+^T cells, macrophages, monocytes, etc.) among the high and low CDH23 expression groups. As shown in Fig. 4A, compared to that in the patients with low CDH23 expression, the patients with high CDH23 expression had a higher proportion of monocytes, memory activated CD4^+^T cells, and regulatory T cells, whereas the percentage of naïve B cells, eosinophils, resting mast cells, plasma cells, memory resting CD4^+^T cells, and CD8^+^T cells was lower in patients with low CDH23 expression levels. Other immune cells were not significantly different between the two groups. This result indicated that high and low CDH23 expression patients had differences in immune cell infiltration in the TME, which has potential roles in tumor occurrence, progression, prognosis, and tumor sensitivity to immunotherapy of patients with AML. Additionally, we used the TIMER database to further investigate the role of CDH23 in the TME. Recently, the ESTIMATE algorithm has been widely utilized to assess immune and stromal scores, which provided in-depth insights into the TME of AML. The analysis revealed that average ESTIMATE, immune, and stromal scores (*P*<0.001) of the high CDH23 expression group were higher than those of the low CDH23 expression group, and there was a statistically significant positive correlation between CDH23 expression levels and the immune and stromal scores (Fig. 4B-D, *P*<0.001). Collectively, these results suggested that the expression level of CDH23 was highly correlated with tumor immune infiltration and the TME.

### Functional enrichment analysis of CDH23 and related genes in AML patients

**Fig. 5:**
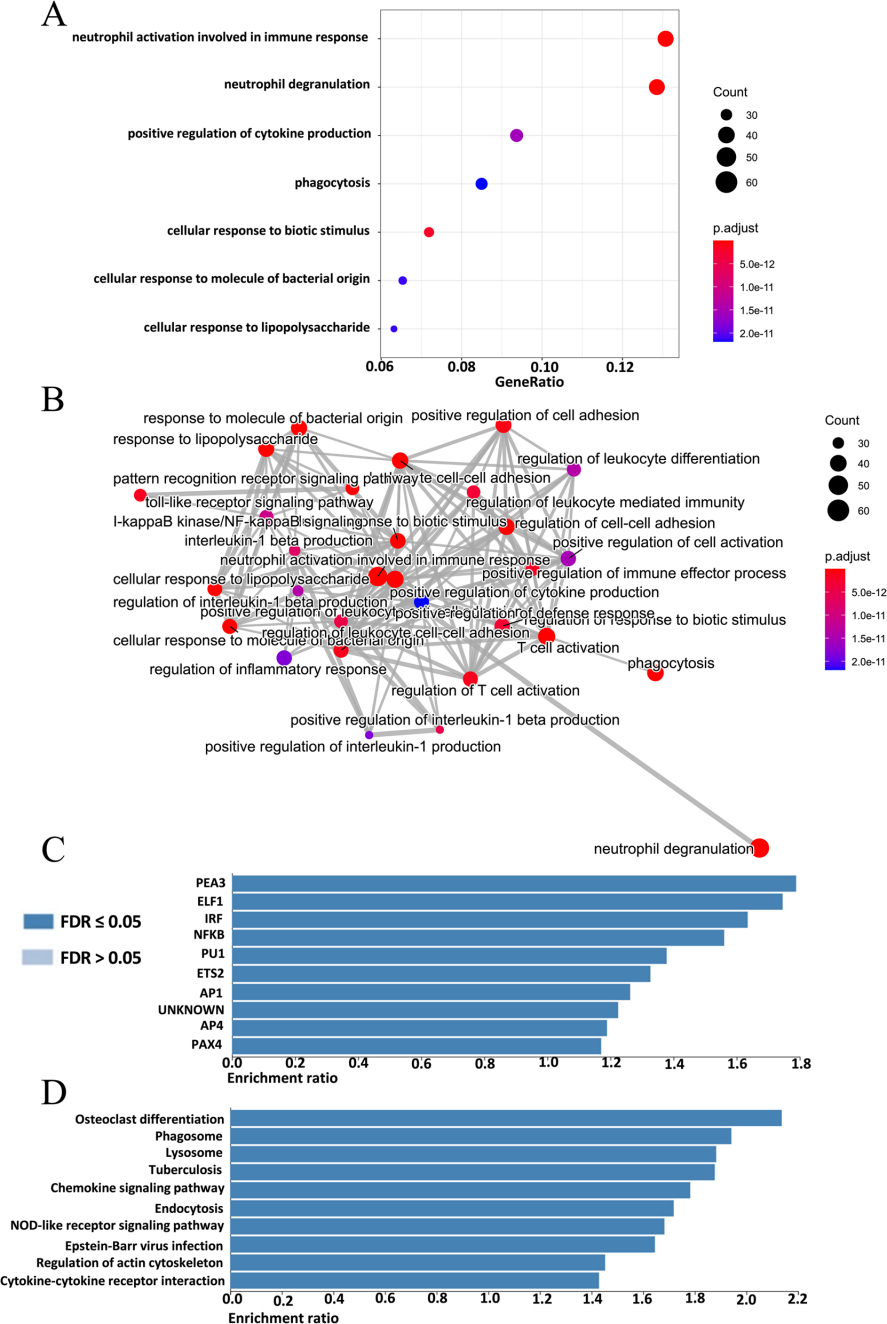
Gene set enrichment analysis and transcription factor target of CDH23. **A-B** Based on the LinkedOmics dataset, we supplied the dotplot and emapplot to perform the GO pathway analysis. **C-D** We also analyzed the transcription factor target and KEGG pathways of CDH23 in AML

To further study the molecular functions and biological processes associated with *CDH23*, we employed the LinkFinder module of the LinkedOmics database to analyze the mRNA sequencing data of patients with AML in TCGA. The GO enrichment analysis data indicated that the expression of *CDH23* and most of the related genes were correlated to pathways or biological processes of immune response and cytokine production such as neutrophil activation involved in immune response, neutrophil degranulation, positive regulation of cytokine production, phagocytosis, cellular response to biotic stimulus, cellular response to molecules of bacterial origin, cellular response to lipopolysaccharide, and so on (Fig. 5A and B). Moreover, the significant transcription factor targets of *CDH23* included *PEA3, ELF1, IRF, PU.1*, and so on (Fig. 5C). Furthermore, the main pathways related to the functions of *CDH23* expression level in AML were illustrated through KEGG analysis and included osteoclast differentiation, phagosome, lysosome, chemokine signaling pathway, endocytosis, NOD-like receptor signaling pathway, regulation of actin cytoskeleton and others (Fig. 5D). In conclusion, CDH23 had an extensive influence on the regulation of several pathways and processes involved in tumor immunity.

### PPI network of CDH23 constructed using GeneMANIA

**Fig. 6:**
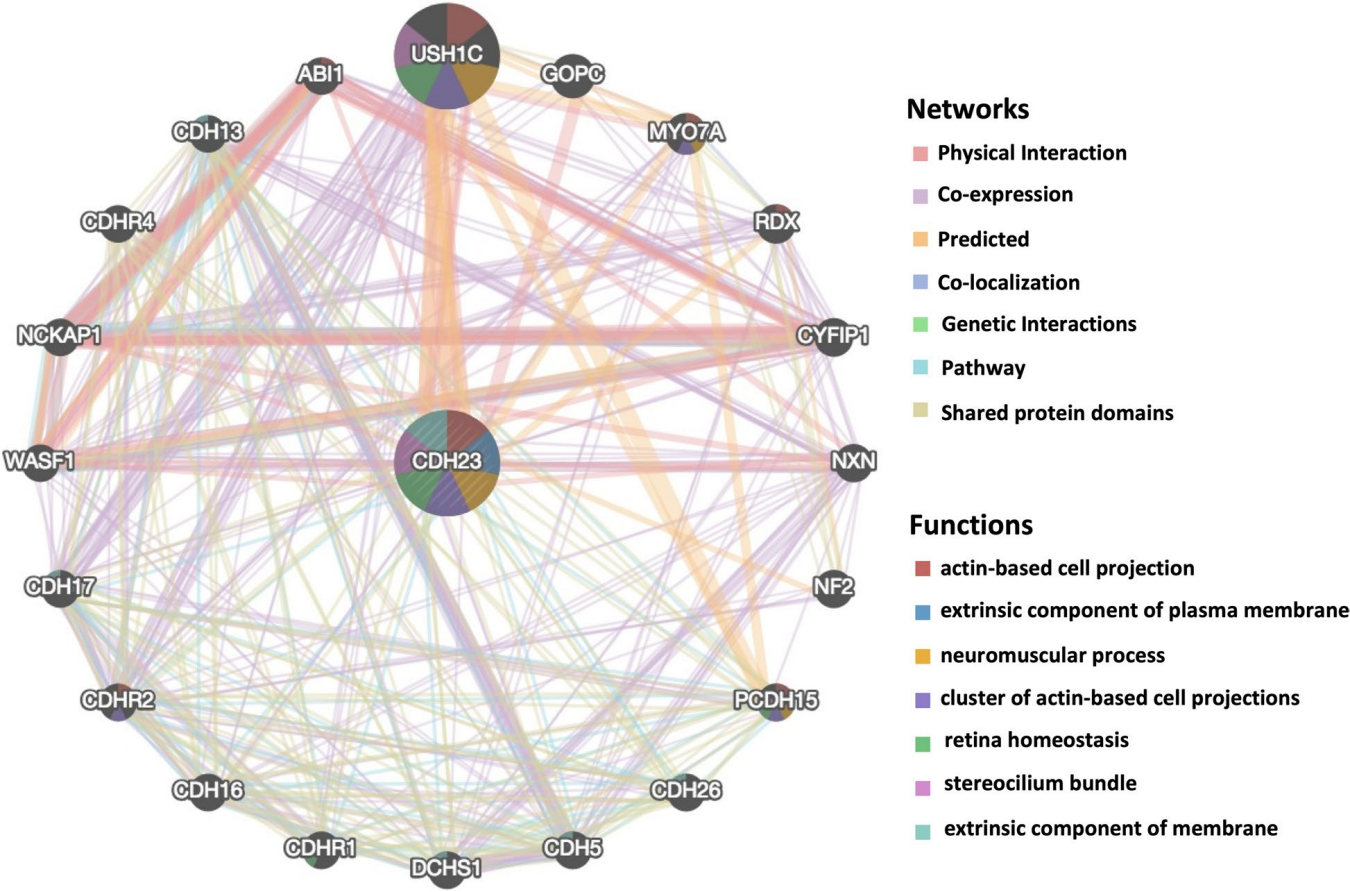
PPI network analysis of CDH23. We used the GeneMANIA database to perform protein-protein interaction of CDH23. The colors of the line illustrate different type of their relationships. The distinct colors inside the gene dots indicate the biological function which these genes involved in, including actin-based cell projection, extrinsic component of plasma membrane, neuromuscular process, cluster of actin-based cell projections, retina homeostasis, stereocilium bundle and extrinsic component of membrane

To further elucidate the role of CDH23 in tumorigenesis, we constructed an integrated PPI network using GeneMANIA to identify CDH23-binding proteins. As described in Fig. 6, the interaction network showed that CDH23 is highly linked with usher syndrome 1C , abl-interactor 1, NCK-associated protein 1 , cytoplasmic FMR1 interacting protein 1, protocadherin-related 15, and other vital proteins. The biological functions of these proteins were related to hearing loss, regulation of actin cytoskeleton, cell adhesion, cancer progression, and so on. Among the interacting proteins, we observed enrichment of biological processes associated with actin-based cell projection, extrinsic components of the plasma membrane, clusters of actin-based cell projections, and extrinsic components of membranes. These results strongly support the hypothesis that CDH23 is involved in the tumorigenesis and pathogenesis of AML.

## Discussion

Although the role of CDH23 in cancer progression and prognosis has been illustrated earlier [18, 27], systematic and comprehensive analysis of its role in AML using bioinformatics tools has not yet been conducted. We first explored the association between *CDH23* mRNA expression level and prognosis of AML patients.

Here, we revealed that *CDH23* expression was higher in AML than in normal counterpart samples. The results showed that higher mRNA expression of *CDH23* corresponded to shorter OS and was determined as an independent predictor of AML patient OS. These findings raise the possibility that CDH23 may serve as a potential prognostic marker for AML.

Furthermore, using gene set enrichment analysis, we identified the most correlated genes, including *ITGAM*, *TFEB*, *PPM1M*, and *SLC8A1*. Notably, *ITGAM*, a CD11 antigen-like family member B (CD11b), encodes the integrin α M chain and plays key roles in regulating various immune cell adhesion and ingestion of coated particles. It also involved in activation, chemotaxis, and cytotoxicity of leukemia cells in the TME, and a number of studies have reported that the prognosis of AML patients with upregulated *ITGAM* expression was poor [28]. The transcription factor EB (*TFEB*) belongs to a member of the basic helix-loop-helix leucine zipper family of transcription activators and acts as the master modulator of lysosomal biogenesis [29]. *TFEB* was associated with tumorigenesis and its elevated expression could promote autophagy and lysosomal biogenesis activating related signaling pathways to control cell proliferation and tumor survival and progression [30]. Taken together, these results highlight the potential ability of these correlated genes to be used as novel prognostic biomarkers and therapeutic targets in the future.

We also investigated the potential correlation between CDH23 expression and tumor immune infiltration in AML. Previous studies have explored the correlations between immune infiltration with prognostic outcome in AML. The proportion of macrophages was reported to be related to the survival and chemotherapy resistance of patients with AML [31]. Regulatory T cells were also revealed to play an important role in occurrence, progression, and immune response of AML [32]. In the present study, we first demonstrated a significant correlation between CDH23 expression and the infiltration level of various immune cells in AML. Notably, high CDH23 expression corresponded with a significantly higher infiltration of monocytes. Accordingly, it has been reported that a higher frequency of monocytes in peripheral blood mononuclear cells is observed in immunotherapy responders compared to non-responders and allows for the prediction of responsiveness prior to the initiation of immunotherapy [33, 34]. These findings indicated that the differences of immune cell infiltration existed between the high and low CDH23 expression patients in the TME, which might provide a guidance for disease outcomes and tumor sensitivity to immunotherapy.

We further explored the functions of *CDH23*, and the results showed that *CDH23* might regulate the pathways associated with osteoclast differentiation, endocytosis, clusters of actin-based cell projections, and so on. Previously, it has been revealed that osteoclasts and osteoblasts are involved in the formation of hematopoietic stem and progenitor cell microenvironment, which provides a support for the subtypes of cells that directly play roles in hematopoiesis and habitat for leukemic blasts [35]. In brief, these findings suggest that *CDH23* may be involved in tumor processes including tumor invasion, phagocytosis, granulocyte activation, and neutrophil-mediated immunity.

Collectively, our bioinformatics analysis systematically disclosed statistical correlations of CDH23 expression with the prognosis and extent of immune cell infiltration of AML. We also comprehensively analyzed the functional pathways of CDH23, which provided bioinformatics and computational biology-based insights for further understanding of the role played by CDH23 in tumor processes.

## Conclusions

In this study, we revealed that *CDH23* was overexpressed in patients with AML. In addition, higher mRNA expression of *CDH23* was found to be related to a reduced OS of AML patients. Additionally, the expression of CDH23 may mediate immune infiltration of tumor. In summary, we have determined that CDH23 might be a potential prognostic biomarker and a promising therapeutic target for AML.

## Supplementary Information

**Additional file 1:** **Fig. S1.** The correlation of CDH23 and blasts percentage. A The different proportion of blast cells between high and low CDH23 mRNA level in TCGA cohort (*P*=0.291). B The spearman correlation analysis between the CDH23 expression level and blast percentage in TCGA cohort (Spearman’s correlation: -0.078, *P*=0.35).

## Data Availability

The datasets supporting the conclusions of this article are available in The Cancer Genome Atlas (https://portal.gdc.cancer.gov/) and the Beat AML trial (ClinicalTrials.gov NCT03013998).

## Abbreviations

AML: Acute myeloid leukemia
OS: Overall survival
CDH23: Cadherin-related 23
HR: Hazard ratio
TME: Tumor microenvironment
TCGA: The Cancer Genome Atlas
GEPIA: Gene Expression Profiling Interactive Analysis
TIMER: Tumor Immune Estimation Resource
GO: Gene Ontology
KEGG: Kyoto Encyclopedia of Genes and Genomes
PPI: Protein-protein interaction
