# Supplementary material for: Role of CDH23 as a prognostic biomarker and its relationship with immune infiltration in acute myeloid leukemia
Source: BMC Cancer. 2022 May 21;22:568. doi: 10.1186/s12885-022-09532-1 (PMC9123811; doi:10.1186/s12885-022-09532-1)

**Additional file 1: Fig. S1** The correlation of CDH23 and blasts percentage. **A** The different proportion of blast cells between high and low CDH23 mRNA level in TCGA cohort (*P*=0.291). **B** The spearman correlation analysis between the CDH23 expression level and blast percentage in TCGA cohort (Spearman’s correlation: -0.078, *P*=0.35).


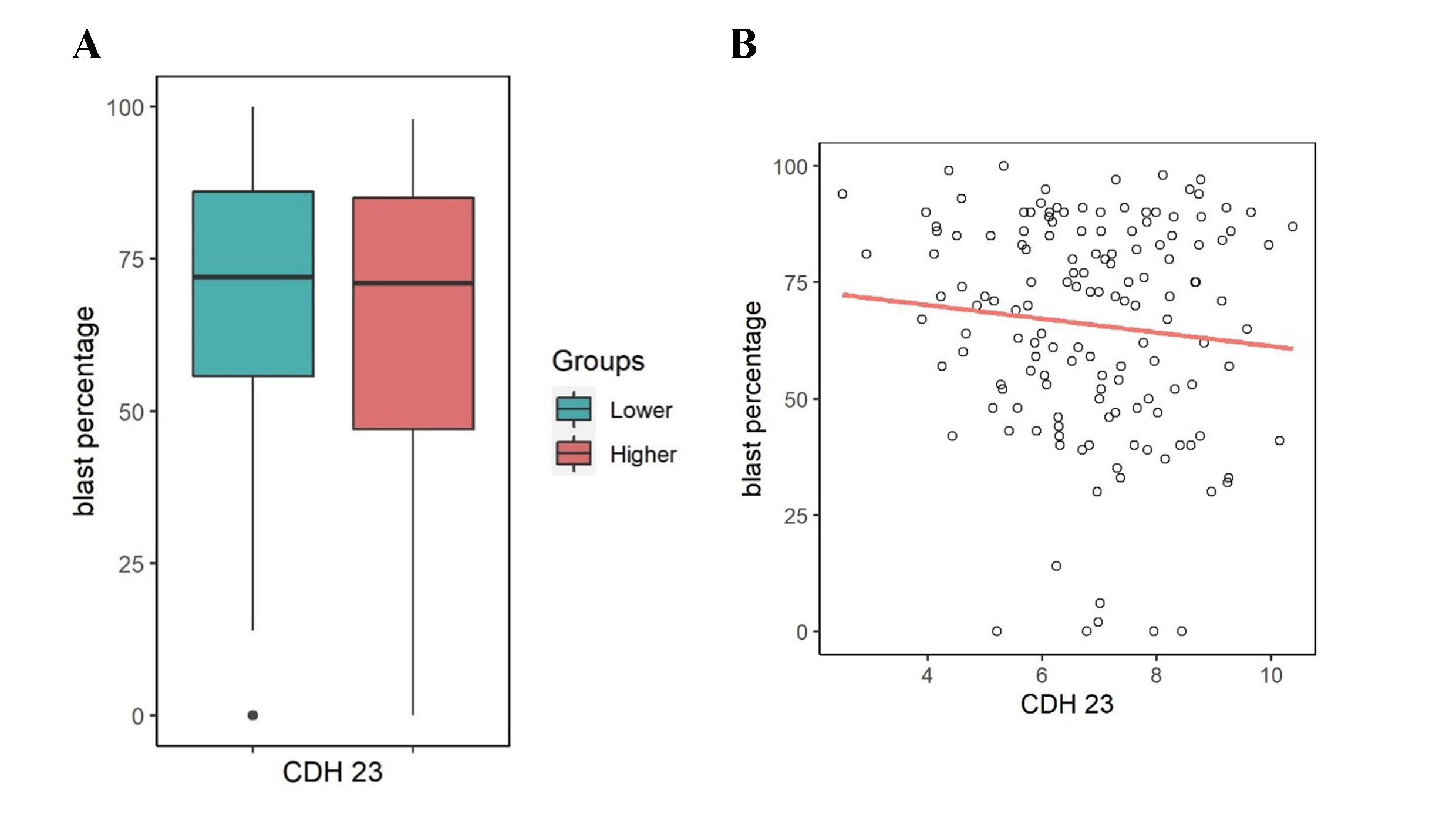

Supplement: Supplementary file 1 — Additional file 1: Fig. S1. The correlation of CDH23 and blasts percentage. A The different proportion of blast cells between high and low CDH23 mRNA level in TCGA cohort (P=0.291). B The spearman correlation analysis between the CDH23 expression level and blast percentage in TCGA cohort (Spearman’s correlation: -0.078, P=0.35). [file 12885_2022_9532_MOESM1_ESM.docx]
